# 9-(2,3-Dichloro­phen­yl)-4a-hydr­oxy-3,3,6,6-tetra­methyl-4,4a,5,6,9,9a-hexa­hydro-3*H*-xanthene-1,8(2*H*,7*H*)-dione

**DOI:** 10.1107/S1600536808002183

**Published:** 2008-01-25

**Authors:** Ghodsi Mohammadi Ziarani, Alireza Abbasi, Alireza Badiei, Mahboubeh Haddadpour, Ali Abdi Jahangir

**Affiliations:** a Department of Chemistry, University of Alzahra, Tehran, Iran; b School of Chemistry, University College of Science, University of Tehran, Tehran, Iran

## Abstract

Mol­ecules of the title compound, C_23_H_26_Cl_2_O_4_, are linked by hydrogen bonds between the hydroxyl O atom and the carbonyl O atom of a neighboring mol­ecule. The central hydropyran and fused cyclohexanone rings adopt half-chair conformations, while the fused hydroxycyclohexanone ring adopts a chair conformation.

## Related literature

For the synthesis of xanthenes, see: Kantevari *et al.* (2006 ▶); Lin *et al.* (2007 ▶). For therapeutic effects, see: Sirkecioglu *et al.* (1995 ▶).
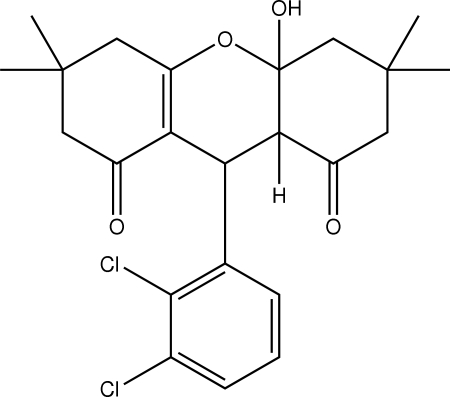


## Experimental

### 

#### Crystal data

C_23_H_26_Cl_2_O_4_
*M* *_r_* = 437.34
Monoclinic, 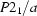

*a* = 11.9581 (17) Å
*b* = 15.165 (2) Å
*c* = 12.3953 (18) Å
β = 105.357 (13)°
*V* = 2167.5 (5) Å^3^
*Z* = 4
Mo *K*α radiation
μ = 0.33 mm^−1^
*T* = 290 (2) K
0.22 × 0.10 × 0.09 mm

#### Data collection

Stoe IPDS diffractometer
Absorption correction: numerical (*X-RED*; Stoe & Cie, 1997 ▶) *T*_min_ = 0.930, *T*_max_ = 0.969
13922 measured reflections
4012 independent reflections
1511 reflections with *I* > 2σ(*I*)
*R*_int_ = 0.070

#### Refinement

*R*[*F* ^2^ > 2σ(*F* ^2^)] = 0.060
*wR*(*F* ^2^) = 0.162
*S* = 0.85
4012 reflections
246 parameters
H atoms treated by a mixture of independent and constrained refinement
Δρ_max_ = 0.73 e Å^−3^
Δρ_min_ = −0.83 e Å^−3^

### 

Data collection: *IPDS Software* (Stoe & Cie, 1997 ▶); cell refinement: *IPDS Software*; data reduction: *IPDS Software*; program(s) used to solve structure: *SHELXS97* (Sheldrick, 2008 ▶); program(s) used to refine structure: *SHELXL97* (Sheldrick, 2008 ▶); molecular graphics: *DIAMOND* (Brandenburg, 2001 ▶); software used to prepare material for publication: *PLATON* (Spek, 2003 ▶).

## Supplementary Material

Crystal structure: contains datablocks I, global. DOI: 10.1107/S1600536808002183/ng2413sup1.cif

Structure factors: contains datablocks I. DOI: 10.1107/S1600536808002183/ng2413Isup2.hkl

Additional supplementary materials:  crystallographic information; 3D view; checkCIF report

## Figures and Tables

**Table 1:** Hydrogen-bond geometry (Å, °)

*D*—H⋯*A*	*D*—H	H⋯*A*	*D*⋯*A*	*D*—H⋯*A*
O2—H22⋯O3^i^	0.79 (4)	2.12 (4)	2.879 (4)	160 (5)

Symmetry code: (i) 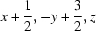.

**Fig. 1.**
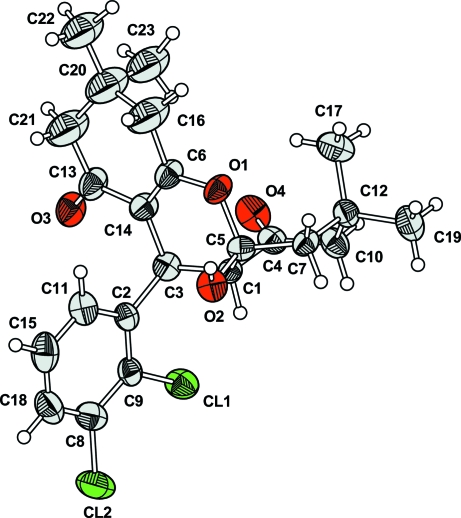
Molecular structure of (I), with 50% probability displacement ellipsoids. H atoms are shown as circles of arbitrary radii.

**Fig. 2.**
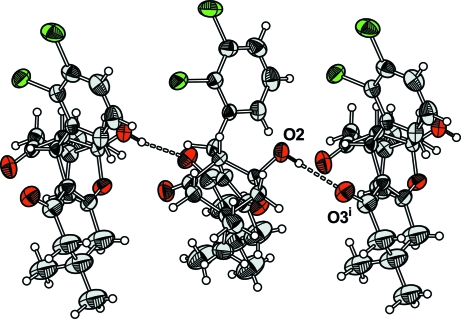
Packing view for (I), showing hydrgen bonding

## supplementary crystallographic information

### Comment

Xanthenes and benzoxanthenes have received much attention because of their wide range of therapeutic and biological properties (Sirkecioglu, *et al.*, 1995). During our study for the synthesis of octahydroquinazolinone (Lin, *et al.*, 2007; Kantevari *et al.*, 2006), we observed that in the reaction conditions, the formation of 1,8-dioxo-octahydroxanthene was occurred in excellent yields in the presence of activated SBA-sulfonic acid as new nanoporous catalyst.

The molecular and atom-labeling scheme for (I) are shwon in Fig.1. The relatively strong, O2—H22···O3, 2.879 (4) Å hydrogen bonds between the neigbouring molecules, seems to be stabilized the crystal structure (Fig. 2). Ring A cyclohex-2-enone (C6/C14/C13/C21/C20/C16) and ring B cyclohexanone (C1/C4/C10/C12/C7/C5) are of course not planar. The C6?C14 can be in resonance with C13?O3, which make the ring A more planar than the ring B. This effect can also be checked by comparing nearly planar torsion angle of O3—C13—C14—C6, 174.1 (4)°, while the torsion angle of O4—C4—C1—C5 found to be -123.8 (4)°.

### Experimental

A mixture of 5,5-dimethyl-1,3-cyclohexanedione (dimedone) (10 mmol, 1.04 g), 2,3-dichlorobenzaldehyde (10 mmol), urea (15 mmol) and activated SBA-sulfonic acid (0.02 g) was heated at 80°C. The reaction was monitored by TLC. After 5 minutes, the reaction was completely solidified. The solid was washed with water and filtered. The crude product was dissolved in hot EtOH and filtered to remove the catalyst. The crystals was appeared after slow cooling. Recystallization did not yield larger crystals.

### Refinement

H atoms were geometrically positioned except hydroxyl group which is located on electron desity map and all constrained to ride on their parent atoms, with *U*_iso_(H) = 1.5*U*_eq_(for methyl group and for the rest 1.2).

### Crystal data

**Table:**

C_23_H_26_Cl_2_O_4_	*F*_000_ = 920
*M**_r_* = 437.34	*D*_x_ = 1.340 Mg m^−^^3^
Monoclinic, *P*2_1_/*a*	Mo *K*α radiation λ = 0.71073 Å
Hall symbol: -P 2yab	Cell parameters from 13922 reflections
*a* = 11.9581 (17) Å	θ = 3.3–25.5º
*b* = 15.165 (2) Å	µ = 0.33 mm^−^^1^
*c* = 12.3953 (18) Å	*T* = 290 (2) K
β = 105.357 (13)º	Block shape, colorless
*V* = 2167.5 (5) Å^3^	0.22 × 0.10 × 0.09 mm
*Z* = 4	

### Data collection

**Table:**

Stoe IPDS diffractometer	4012 independent reflections
Radiation source: fine-focus sealed tube	1511 reflections with *I* > 2σ(*I*)
Monochromator: graphite	*R*_int_ = 0.070
*T* = 290(2) K	θ_max_ = 25.5º
φ oscillation scans	θ_min_ = 3.7º
Absorption correction: numerical(X-RED; Stoe & Cie, 1997)	*h* = −14→12
*T*_min_ = 0.930, *T*_max_ = 0.969	*k* = −17→18
13922 measured reflections	*l* = −15→15

### Refinement

**Table:**

Refinement on *F*^2^	Hydrogen site location: inferred from neighbouring sites
Least-squares matrix: full	H atoms treated by a mixture of independent and constrained refinement
*R*[*F*^2^ > 2σ(*F*^2^)] = 0.060	*w* = 1/[σ^2^(*F*_o_^2^) + (0.08*P*)^2^] where *P* = (*F*_o_^2^ + 2*F*_c_^2^)/3
*wR*(*F*^2^) = 0.162	(Δ/σ)_max_ < 0.001
*S* = 0.85	Δρ_max_ = 0.73 e Å^−^^3^
4012 reflections	Δρ_min_ = −0.83 e Å^−^^3^
246 parameters	Extinction correction: SHELXL97 (Sheldrick, 2008), Fc^*^=kFc[1+0.001xFc^2^λ^3^/sin(2θ)]^-1/4^
Primary atom site location: structure-invariant direct methods	Extinction coefficient: 0.0056 (13)
Secondary atom site location: difference Fourier map	

### Special details

**Table:**

Geometry. All e.s.d.'s (except the e.s.d. in the dihedral angle between two l.s. planes) are estimated using the full covariance matrix. The cell e.s.d.'s are taken into account individually in the estimation of e.s.d.'s in distances, angles and torsion angles; correlations between e.s.d.'s in cell parameters are only used when they are defined by crystal symmetry. An approximate (isotropic) treatment of cell e.s.d.'s is used for estimating e.s.d.'s involving l.s. planes.
Refinement. Refinement of *F*^2^ against ALL reflections. The weighted *R*-factor *wR* and goodness of fit *S* are based on *F*^2^, conventional *R*-factors *R* are based on *F*, with *F* set to zero for negative *F*^2^. The threshold expression of *F*^2^ > σ(*F*^2^) is used only for calculating *R*-factors(gt) *etc*. and is not relevant to the choice of reflections for refinement. *R*-factors based on *F*^2^ are statistically about twice as large as those based on *F*, and *R*- factors based on ALL data will be even larger.

### Fractional atomic coordinates and isotropic or equivalent isotropic displacement parameters (Å^2^)

**Table:**

	*x*	*y*	*z*	*U*_iso_*/*U*_eq_	
Cl1	0.74989 (9)	0.77065 (8)	−0.11681 (10)	0.0535 (4)	
Cl2	0.80090 (12)	0.65780 (9)	−0.30989 (10)	0.0694 (5)	
O1	1.1085 (2)	0.84256 (18)	0.2731 (2)	0.0445 (8)	
O2	1.1313 (2)	0.8477 (2)	0.0944 (3)	0.0469 (9)	
H22	1.197 (4)	0.851 (3)	0.130 (4)	0.056*	
O3	0.8621 (3)	0.59442 (19)	0.2062 (3)	0.0532 (9)	
O4	0.7952 (3)	0.8634 (2)	0.2112 (3)	0.0571 (9)	
C1	0.9390 (3)	0.8517 (3)	0.1106 (3)	0.0315 (10)	
H1	0.9188	0.8699	0.0319	0.038*	
C2	0.9459 (3)	0.7008 (3)	0.0160 (4)	0.0337 (11)	
C3	0.9198 (3)	0.7533 (3)	0.1118 (3)	0.0365 (11)	
H3	0.8371	0.7449	0.1056	0.044*	
C4	0.8591 (4)	0.9029 (3)	0.1672 (4)	0.0398 (11)	
C5	1.0648 (3)	0.8800 (3)	0.1620 (4)	0.0347 (11)	
C6	1.0712 (4)	0.7622 (3)	0.2944 (4)	0.0415 (11)	
C7	1.0802 (3)	0.9788 (3)	0.1793 (3)	0.0367 (11)	
H7A	1.0751	1.0057	0.1071	0.044*	
H7B	1.1580	0.9894	0.2259	0.044*	
C8	0.8930 (4)	0.6534 (3)	−0.1764 (4)	0.0487 (13)	
C9	0.8717 (4)	0.7044 (3)	−0.0908 (4)	0.0370 (11)	
C10	0.8714 (4)	0.9994 (3)	0.1684 (4)	0.0447 (12)	
H10A	0.8157	1.0256	0.2034	0.054*	
H10B	0.8549	1.0211	0.0922	0.054*	
C11	1.0414 (4)	0.6451 (3)	0.0339 (4)	0.0473 (12)	
H11	1.0932	0.6423	0.1045	0.057*	
C12	0.9942 (4)	1.0271 (3)	0.2326 (4)	0.0397 (11)	
C13	0.9474 (4)	0.6348 (3)	0.2614 (4)	0.0507 (13)	
C14	0.9840 (4)	0.7182 (3)	0.2255 (4)	0.0408 (11)	
C15	1.0604 (4)	0.5943 (3)	−0.0507 (5)	0.0589 (14)	
H15	1.1234	0.5560	−0.0362	0.071*	
C16	1.1378 (5)	0.7305 (4)	0.4066 (5)	0.0905 (10)	
H16A	1.2129	0.7097	0.4012	0.109*	
H16B	1.1513	0.7801	0.4578	0.109*	
C17	1.0136 (4)	1.0051 (3)	0.3568 (4)	0.0621 (15)	
H17A	0.9625	1.0401	0.3874	0.093*	
H17B	1.0926	1.0176	0.3961	0.093*	
H17C	0.9978	0.9437	0.3648	0.093*	
C18	0.9873 (5)	0.5992 (3)	−0.1572 (5)	0.0559 (14)	
H18	1.0021	0.5661	−0.2151	0.067*	
C19	1.0103 (4)	1.1263 (3)	0.2207 (4)	0.0597 (14)	
H19A	0.9918	1.1416	0.1428	0.090*	
H19B	1.0893	1.1419	0.2559	0.090*	
H19C	0.9598	1.1576	0.2559	0.090*	
C20	1.0817 (5)	0.6589 (4)	0.4551 (5)	0.0905 (10)	
C21	1.0208 (5)	0.5970 (4)	0.3675 (4)	0.0905 (10)	
H21A	0.9719	0.5596	0.3993	0.109*	
H21B	1.0785	0.5593	0.3489	0.109*	
C22	1.1532 (5)	0.6224 (4)	0.5598 (5)	0.0905 (10)	
H22A	1.2121	0.5851	0.5443	0.136*	
H22B	1.1055	0.5884	0.5955	0.136*	
H22C	1.1891	0.6696	0.6083	0.136*	
C23	0.9799 (5)	0.7069 (4)	0.4916 (5)	0.0905 (10)	
H23A	0.9306	0.6636	0.5120	0.136*	
H23B	0.9354	0.7416	0.4303	0.136*	
H23C	1.0119	0.7446	0.5544	0.136*	

### Atomic displacement parameters (Å^2^)

**Table:**

	*U*^11^	*U*^22^	*U*^33^	*U*^12^	*U*^13^	*U*^23^
Cl1	0.0437 (7)	0.0653 (9)	0.0458 (8)	0.0066 (6)	0.0019 (6)	−0.0049 (6)
Cl2	0.0873 (10)	0.0793 (10)	0.0415 (8)	−0.0153 (8)	0.0171 (7)	−0.0107 (7)
O1	0.0449 (18)	0.0365 (19)	0.045 (2)	−0.0107 (15)	−0.0013 (15)	0.0099 (15)
O2	0.0329 (18)	0.050 (2)	0.058 (2)	0.0001 (17)	0.0121 (16)	−0.0025 (17)
O3	0.049 (2)	0.0415 (19)	0.063 (2)	−0.0143 (16)	0.0030 (17)	0.0017 (16)
O4	0.044 (2)	0.063 (2)	0.070 (2)	−0.0123 (17)	0.0261 (18)	−0.0110 (18)
C1	0.030 (2)	0.030 (3)	0.031 (2)	−0.002 (2)	0.0023 (19)	0.001 (2)
C2	0.030 (2)	0.031 (3)	0.041 (3)	−0.006 (2)	0.010 (2)	−0.005 (2)
C3	0.026 (2)	0.040 (3)	0.043 (3)	−0.006 (2)	0.006 (2)	−0.001 (2)
C4	0.032 (3)	0.045 (3)	0.039 (3)	−0.003 (2)	0.003 (2)	−0.006 (2)
C5	0.030 (3)	0.042 (3)	0.033 (3)	−0.001 (2)	0.012 (2)	0.001 (2)
C6	0.035 (3)	0.040 (3)	0.044 (3)	−0.004 (2)	0.001 (2)	0.008 (2)
C7	0.036 (3)	0.035 (3)	0.037 (3)	−0.003 (2)	0.007 (2)	0.006 (2)
C8	0.055 (3)	0.050 (3)	0.042 (3)	−0.020 (3)	0.016 (3)	−0.005 (3)
C9	0.039 (3)	0.030 (3)	0.043 (3)	−0.006 (2)	0.012 (2)	−0.004 (2)
C10	0.040 (3)	0.047 (3)	0.048 (3)	0.007 (2)	0.014 (2)	−0.010 (2)
C11	0.038 (3)	0.045 (3)	0.061 (3)	−0.001 (2)	0.015 (2)	−0.005 (3)
C12	0.044 (3)	0.035 (3)	0.041 (3)	−0.003 (2)	0.014 (2)	−0.005 (2)
C13	0.051 (3)	0.038 (3)	0.056 (3)	−0.011 (3)	0.002 (3)	0.007 (2)
C14	0.037 (3)	0.041 (3)	0.039 (3)	−0.008 (2)	0.002 (2)	0.003 (2)
C15	0.052 (3)	0.043 (3)	0.088 (5)	0.008 (2)	0.029 (3)	−0.001 (3)
C16	0.098 (2)	0.086 (2)	0.0683 (19)	−0.0274 (16)	−0.0119 (15)	0.0250 (15)
C17	0.068 (3)	0.074 (4)	0.046 (3)	−0.017 (3)	0.019 (3)	−0.012 (3)
C18	0.067 (4)	0.047 (3)	0.061 (4)	−0.002 (3)	0.029 (3)	−0.013 (3)
C19	0.071 (4)	0.045 (3)	0.067 (4)	−0.002 (3)	0.026 (3)	−0.012 (3)
C20	0.098 (2)	0.086 (2)	0.0683 (19)	−0.0274 (16)	−0.0119 (15)	0.0250 (15)
C21	0.098 (2)	0.086 (2)	0.0683 (19)	−0.0274 (16)	−0.0119 (15)	0.0250 (15)
C22	0.098 (2)	0.086 (2)	0.0683 (19)	−0.0274 (16)	−0.0119 (15)	0.0250 (15)
C23	0.098 (2)	0.086 (2)	0.0683 (19)	−0.0274 (16)	−0.0119 (15)	0.0250 (15)

### Geometric parameters (Å, °)

**Table:**

Cl1—C9	1.728 (4)	C11—C15	1.368 (6)
Cl2—C8	1.729 (5)	C11—H11	0.9300
O1—C6	1.347 (5)	C12—C19	1.529 (6)
O1—C5	1.453 (5)	C12—C17	1.532 (6)
O2—C5	1.388 (5)	C13—C14	1.447 (6)
O2—H22	0.79 (4)	C13—C21	1.489 (6)
O3—C13	1.230 (5)	C15—C18	1.379 (6)
O4—C4	1.209 (5)	C15—H15	0.9300
C1—C3	1.510 (5)	C16—C20	1.485 (7)
C1—C5	1.532 (5)	C16—H16A	0.9700
C1—C4	1.538 (6)	C16—H16B	0.9700
C1—H1	0.9800	C17—H17A	0.9600
C2—C9	1.386 (6)	C17—H17B	0.9600
C2—C11	1.390 (5)	C17—H17C	0.9600
C2—C3	1.529 (6)	C18—H18	0.9300
C3—C14	1.511 (5)	C19—H19A	0.9600
C3—H3	0.9800	C19—H19B	0.9600
C4—C10	1.470 (6)	C19—H19C	0.9600
C5—C7	1.517 (5)	C20—C22	1.460 (7)
C6—C14	1.338 (5)	C20—C21	1.473 (7)
C6—C16	1.488 (6)	C20—C23	1.584 (8)
C7—C12	1.546 (5)	C21—H21A	0.9700
C7—H7A	0.9700	C21—H21B	0.9700
C7—H7B	0.9700	C22—H22A	0.9600
C8—C18	1.364 (6)	C22—H22B	0.9600
C8—C9	1.390 (6)	C22—H22C	0.9600
C10—C12	1.532 (5)	C23—H23A	0.9600
C10—H10A	0.9700	C23—H23B	0.9600
C10—H10B	0.9700	C23—H23C	0.9600
			
C6—O1—C5	119.0 (3)	C17—C12—C7	112.7 (3)
C5—O2—H22	106 (3)	O3—C13—C14	122.5 (4)
C3—C1—C5	114.1 (3)	O3—C13—C21	120.5 (4)
C3—C1—C4	112.4 (3)	C14—C13—C21	116.9 (4)
C5—C1—C4	109.2 (3)	C6—C14—C13	119.2 (4)
C3—C1—H1	106.9	C6—C14—C3	122.5 (4)
C5—C1—H1	106.9	C13—C14—C3	118.3 (4)
C4—C1—H1	106.9	C11—C15—C18	120.8 (5)
C9—C2—C11	117.9 (4)	C11—C15—H15	119.6
C9—C2—C3	120.7 (4)	C18—C15—H15	119.6
C11—C2—C3	121.3 (4)	C20—C16—C6	115.4 (4)
C1—C3—C14	108.4 (3)	C20—C16—H16A	108.4
C1—C3—C2	116.3 (3)	C6—C16—H16A	108.4
C14—C3—C2	112.5 (3)	C20—C16—H16B	108.4
C1—C3—H3	106.3	C6—C16—H16B	108.4
C14—C3—H3	106.3	H16A—C16—H16B	107.5
C2—C3—H3	106.3	C12—C17—H17A	109.5
O4—C4—C10	124.2 (4)	C12—C17—H17B	109.5
O4—C4—C1	119.9 (4)	H17A—C17—H17B	109.5
C10—C4—C1	115.8 (4)	C12—C17—H17C	109.5
O2—C5—O1	108.3 (3)	H17A—C17—H17C	109.5
O2—C5—C7	111.5 (3)	H17B—C17—H17C	109.5
O1—C5—C7	104.6 (3)	C8—C18—C15	119.0 (4)
O2—C5—C1	107.8 (3)	C8—C18—H18	120.5
O1—C5—C1	110.6 (3)	C15—C18—H18	120.5
C7—C5—C1	113.9 (3)	C12—C19—H19A	109.5
C14—C6—O1	124.7 (4)	C12—C19—H19B	109.5
C14—C6—C16	124.7 (4)	H19A—C19—H19B	109.5
O1—C6—C16	110.6 (4)	C12—C19—H19C	109.5
C5—C7—C12	117.2 (3)	H19A—C19—H19C	109.5
C5—C7—H7A	108.0	H19B—C19—H19C	109.5
C12—C7—H7A	108.0	C22—C20—C21	118.2 (5)
C5—C7—H7B	108.0	C22—C20—C16	114.6 (5)
C12—C7—H7B	108.0	C21—C20—C16	110.8 (5)
H7A—C7—H7B	107.2	C22—C20—C23	103.5 (5)
C18—C8—C9	120.8 (4)	C21—C20—C23	103.6 (5)
C18—C8—Cl2	118.5 (4)	C16—C20—C23	104.1 (5)
C9—C8—Cl2	120.6 (4)	C20—C21—C13	117.8 (5)
C2—C9—C8	120.4 (4)	C20—C21—H21A	107.9
C2—C9—Cl1	119.7 (3)	C13—C21—H21A	107.9
C8—C9—Cl1	119.8 (4)	C20—C21—H21B	107.9
C4—C10—C12	111.0 (4)	C13—C21—H21B	107.9
C4—C10—H10A	109.4	H21A—C21—H21B	107.2
C12—C10—H10A	109.4	C20—C22—H22A	109.5
C4—C10—H10B	109.4	C20—C22—H22B	109.5
C12—C10—H10B	109.4	H22A—C22—H22B	109.5
H10A—C10—H10B	108.0	C20—C22—H22C	109.5
C15—C11—C2	121.0 (4)	H22A—C22—H22C	109.5
C15—C11—H11	119.5	H22B—C22—H22C	109.5
C2—C11—H11	119.5	C20—C23—H23A	109.5
C19—C12—C10	110.3 (4)	C20—C23—H23B	109.5
C19—C12—C17	108.8 (4)	H23A—C23—H23B	109.5
C10—C12—C17	109.4 (4)	C20—C23—H23C	109.5
C19—C12—C7	108.0 (3)	H23A—C23—H23C	109.5
C10—C12—C7	107.7 (3)	H23B—C23—H23C	109.5
			
C5—C1—C3—C14	44.4 (5)	C1—C4—C10—C12	−61.1 (5)
C4—C1—C3—C14	−80.6 (4)	C9—C2—C11—C15	−1.1 (6)
C5—C1—C3—C2	−83.6 (4)	C3—C2—C11—C15	176.0 (4)
C4—C1—C3—C2	151.5 (3)	C4—C10—C12—C19	172.9 (4)
C9—C2—C3—C1	−73.9 (5)	C4—C10—C12—C17	−67.5 (5)
C11—C2—C3—C1	109.1 (4)	C4—C10—C12—C7	55.2 (5)
C9—C2—C3—C14	160.2 (4)	C5—C7—C12—C19	−169.0 (4)
C11—C2—C3—C14	−16.8 (5)	C5—C7—C12—C10	−49.8 (5)
C3—C1—C4—O4	3.8 (5)	C5—C7—C12—C17	70.8 (5)
C5—C1—C4—O4	−123.8 (4)	O1—C6—C14—C13	−175.9 (4)
C3—C1—C4—C10	−179.4 (3)	C16—C6—C14—C13	1.9 (7)
C5—C1—C4—C10	53.0 (5)	O1—C6—C14—C3	2.6 (7)
C6—O1—C5—O2	−84.3 (4)	C16—C6—C14—C3	−179.6 (5)
C6—O1—C5—C7	156.7 (3)	O3—C13—C14—C6	174.1 (4)
C6—O1—C5—C1	33.6 (5)	C21—C13—C14—C6	−8.4 (7)
C3—C1—C5—O2	65.9 (4)	O3—C13—C14—C3	−4.6 (7)
C4—C1—C5—O2	−167.5 (3)	C21—C13—C14—C3	173.0 (5)
C3—C1—C5—O1	−52.3 (5)	C1—C3—C14—C6	−20.3 (6)
C4—C1—C5—O1	74.3 (4)	C2—C3—C14—C6	109.8 (5)
C3—C1—C5—C7	−169.8 (3)	C1—C3—C14—C13	158.3 (4)
C4—C1—C5—C7	−43.2 (5)	C2—C3—C14—C13	−71.6 (5)
C5—O1—C6—C14	−9.9 (6)	C2—C11—C15—C18	2.2 (7)
C5—O1—C6—C16	172.1 (4)	C14—C6—C16—C20	−17.4 (9)
O2—C5—C7—C12	168.1 (3)	O1—C6—C16—C20	160.6 (5)
O1—C5—C7—C12	−75.0 (4)	C9—C8—C18—C15	1.4 (7)
C1—C5—C7—C12	45.9 (5)	Cl2—C8—C18—C15	−179.2 (4)
C11—C2—C9—C8	0.2 (6)	C11—C15—C18—C8	−2.3 (7)
C3—C2—C9—C8	−176.9 (4)	C6—C16—C20—C22	174.0 (6)
C11—C2—C9—Cl1	178.5 (3)	C6—C16—C20—C21	37.2 (8)
C3—C2—C9—Cl1	1.4 (5)	C6—C16—C20—C23	−73.6 (6)
C18—C8—C9—C2	−0.3 (7)	C22—C20—C21—C13	179.6 (5)
Cl2—C8—C9—C2	−179.8 (3)	C16—C20—C21—C13	−45.3 (8)
C18—C8—C9—Cl1	−178.6 (3)	C23—C20—C21—C13	65.8 (6)
Cl2—C8—C9—Cl1	1.9 (5)	O3—C13—C21—C20	−150.8 (5)
O4—C4—C10—C12	115.6 (5)	C14—C13—C21—C20	31.5 (8)

### Hydrogen-bond geometry (Å, °)

**Table:**

*D*—H···*A*	*D*—H	H···*A*	*D*···*A*	*D*—H···*A*
O2—H22···O3^i^	0.79 (4)	2.12 (4)	2.879 (4)	160 (5)

Symmetry codes: (i) *x*+1/2, −*y*+3/2, *z*.
